# Hot fomentation of newborn fontanelles as an indigenous practice in Ghana: implications for policy and integrated community-based health care in Covid-19 pandemic and beyond

**DOI:** 10.1186/s12939-023-01852-3

**Published:** 2023-02-27

**Authors:** Mary Ani-Amponsah, Solina Richter, Mariam Al-Hassan Adam, Evans Appiah Osei, Mahama Mustapha, Ezekiel Oti-Boadi

**Affiliations:** 1 Maternal & Child Health Department, School of Nursing and Midwifery, University of Ghana, Legon, Accra, Ghana; 2 Faculty of Nursing, ECHA 5-2381, Edmonton Clinic Health Academy (ECHA), University of Alberta, 11405 87 Avenue, Edmonton, T6G 1C9 Canada; 3 Out-Patient Department, Ashaiman Polyclinic, Ghana Health Service, Accra, Ghana; 4 Purdue University, West Lafayette, USA; 5 Department of Child Health, Tamale Teaching Hospital, Tamale, Northern Region Ghana; 6 Department of Nursing, School of Nursing and Midwifery, Valley View University, Accra, Ghana

**Keywords:** Newborn, Hot fontanelle fomentation, Indigenous practice, Ghana, Covid-19

## Abstract

**Objective:**

African newborns undergo numerous traditional and religious practices ranging from fontanelle fomentation to total head shaving, scalp molding, skin scarification and ano-genital irrigation which can negatively impact the health of neonates. Hot fomentation of fontanelles has been a predominant indigenous home-based postnatal practice in Ghana and among Africans in the diaspora. Mobility restrictions during the Covid-19 pandemic has impacted direct access to facility-based care as well as home care. The flourishing of newborn traditional practices among African populations during this Covid-19 pandemic offers opportunities to rethink the provision of family healthcare support for newborns during the ongoing pandemic and beyond. Hence, the aim of this critical review was to examine and describe a common indigenous practice—hot fontanelle fomentation to inform home birth support, discharge planning, and the delivery of optimal home-based care support.

**Study design:**

This study is a review of literature on hot fomentation of newborn fontanelles.

**Methods:**

Literature search in CINAHL, PubMed, African Index Medicus and Scopus, was conducted and evidence synthesised from articles ranging from 1983–2022. Sixty articles were reviewed; however, 10 manuscripts were excluded prior to screening. The other 19 were exempted because they were either below 1983 or were not the best fit for the study purpose. In all, 31 studies were included in the study. The study was guided by Madeleine Leininger’s Culture Care Diversity and Universality care theory.

**Results:**

The current study identifies hot fomentation of newborn fontanelles practices in Ghana, the description of hot fomentation practices and the dangers associated with it. The findings and suggested ways to help overcome this challenge.

**Conclusion:**

There are several neonatal indigenous practices including fontanelle fomentation which pose threat to the health of the neonate as discussed in this study. Future research needs to investigate innovative ways of fontanelle fomentation where necessary instead of the use of hot water by mothers, especially in this Covid-19 pandemic where health and mobility restrictions impact physical access to timely health care. This research will help educate mothers about the dangers of fontanel fomentation and reduce the practice, especially in rural areas of developing countries. This could help reduce neonatal mortality and unnecessary healthcare costs.

## Introduction

Since December 2019, the COVID-19 pandemic, caused by the novel coronavirus, has had a significant global impact, affecting countries such as China, Italy, Spain, the United States, and the United Kingdom [1, 2]. The pandemic has posed challenges to health systems and emergency response systems worldwide in managing the disease's morbidity and mortality [1]. In Africa, COVID-19-related restrictions on mobility have adversely affected the health and access to healthcare, especially for vulnerable populations [2, 3]. The Covid-19 restrictions have significantly impacted maternal and newborn healthcare delivery with subsequent effects on newborn care post-delivery [4]. Traditional health practices for newborn care, such as head molding, nasal administration of local medicines, ano-genital irrigation, and hot fomentation of fontanelles, are commonly provided by families [5]. The dissemination of indigenous newborn care practices is primarily done through practical demonstrations and oration by traditional knowledge holders and observers. However, despite being widely practiced, there is a lack of literature on traditional practices, including among Africans in the diaspora. This critical review will examine the high-risk indigenous practice of fontanelle fomentation on newborns in Ghana.

### Description of the newborn fontanelle

The newborn anterior fontanelle (NAF), also known as the bregmatic fontanelle, or frontal fontanelle is a diamond or rhomboid-shaped structural membranous space, measuring about 4 cm in its antero-posterior dimension and 2.5 cm in its transverse diameter [6, 7]. The word “fontanelle” is derived from the Latin word ‘fonticulus’ and the French word ‘*fontaine*’, meaning a little fountain or natural spring [8]. The NAF is located between the two frontal and two parietal bones of the developing skull, and it is bordered by the junctions of the frontal, coronal and sagittal sutures with its membranous normotensive structure. The most common causes of a large anterior fontanelle or delayed fontanelle closure are achondroplasia, hypothyroidism, Down syndrome, increased intracranial pressure, infections, dehydration and rickets [9, 10]. The anterior fontanelle persists until approximately 18 months, when full closure is observed [11]. The molding of the fontanelles aid in delivery through the birth canal and it usually resolves after three to four days of birth. In clinical assessment, the NAF is palpated as part of all newborn and infant physical examinations (NIPE); its measurement needs to be performed as an average of the anterior–posterior and the transverse measurements [12].

There are six sensitive membranous anatomical structures that require delicate care during delivery and after birth, with the NAF being the most prominent. These structures consist of one anterior, one posterior, two sphenoid, and two mastoids [10]. After birth, the six fontanelles play a crucial role in aiding the brain's expansion. The triangular-shaped posterior fontanelle, located at the junction of the occipital and two parietal bones, helps in the careful maneuvering of the newborn during delivery by providing support to the soft sutures, allowing for accommodation of passage through the birth canal [12]. The anterior fontanelle of the newborn is the largest of the six fontanelles which support delivery, brain expansion and development, and thermoregulation, but it receives the most pressure of heat during the hot fomentation [13].

In Africa, including Egypt, traditional art forms such as carvings, craftworks, and verbal artistry have a strong presence and are more prevalent than popular writing traditions, especially concerning community practices and the communication of our African identity [14–16]. Upon the birth of a newborn, the paternal family expresses gratitude to the mother for bringing pride and honor to the family. The presence of the newborn reinforces marital and family bonds. Traditionally, the primary responsibility of caring for the newborn falls under the purview of the paternal family, with grandmothers, sisters-in-law, and aunties playing a vital role in the child's physical care. Female adults, particularly mother-in-laws and grandmothers, actively participate in the newborn's physical care for 2 to 3 months after delivery. Traditional practices such as bathing the newborn with herbs to boost their immune system and protect them from illnesses and the evil eye are common. Additionally, throwing the baby into the air and catching them is sometimes done to instill bravery and resilience in the child. During traditional naming ceremonies, it is customary to touch the newborn's tongue with alcohol and water sequentially to symbolically introduce the child to truth telling and falsehood, considered essential for growth and development.

### Indigenous newborn practice

Harmful indigenous newborn practices are noted to set neonates on challenging health trajectories [17–19]. The practice of fontanelle fomentation poses health threats to the newborn; however, its respective dangers have least been examined or discussed over the years. In other African countries such as Nigeria, abdominal scarification with razor blades following febrile illnesses is a traditional neonatal practice that sometimes lead to evisceration of intra-abdominal viscera [20]. In rural Uganda, false tooth extraction performed by traditional healers to resolve fever and diarrhoea is associated with anaemia and septicaemia [21]. The use of Mahogany and Neem oil (Bornu) to the newborn fontanelle and head molding are known practices in Nigeria but their impact on child growth and development have not been examined [17, 22].

## Methods

This study is a review of literature on hot fomentation of newborn fontanelles. A literature search in CINAHL, PubMed, African Index Medicus and Scopus, was conducted and evidence was synthesized from articles ranging from 1983–2022.

**Fig. 1:**
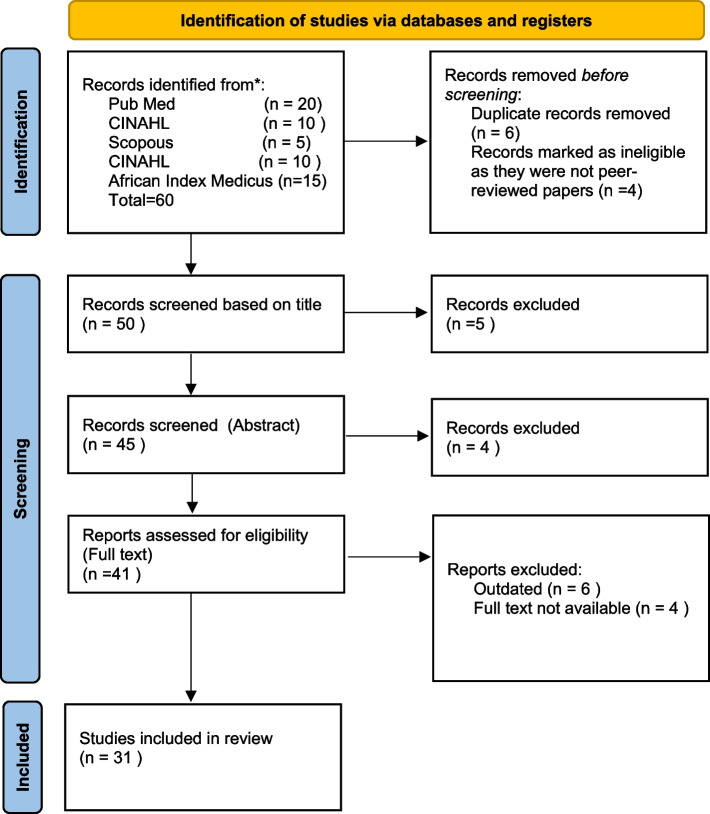
Prisma flow diagram for study inclusion

This paper utilized quantitative studies, qualitative studies, and a systematic literature review. Initially, about 60 studies were assessed, but only 31 were used for this write-up. Ten studies were excluded due to duplication and lack of peer review, while 19 studies were deemed unsuitable for the study's purpose or dated before 1983. The PRISMA FLOW in diagram 1 provides a visual representation of the criteria and rationale for excluding studies. The paper's search for information and inclusion of studies utilized the keywords Newborn, Hot Fontanelle Fomentation, Indigenous Practice, Ghana, and Covid-19. The details of the criteria for inclusion is in Fig. 1 below.

## Results

### Understanding fontanelle fomentation

In Ghana, Akans form the largest ethnic group (47.5%), followed by the Mole Dagbani (16.6%), the Ewes (13.9%), Ga-Dangme (7.4%), and the Mande population (1.1%) [23]. Fontanelle fomentation (FF) is a predominant customary practice among these ethnic groups in Ghana and Africans in the diaspora [24]. As a part of home-based postnatal care in Ghana, newborns receive hot fomentation of the fontanelles and scalp molding to achieve a round head shape, which is considered typical of Ghanaians. The practice of newborn fontanelle fomentation (NFF), especially of the anterior fontanelle, is based on the belief that this sensitive area of the newborn's head requires healing assistance. Moist heat is applied to speed up bone ossification and promote fontanelle closure by improving blood circulation [24].

The sunken and late closure of newborn fontanelle has been associated with ‘*asram*’- the local name for a fatal newborn illness that is characterized by head enlargement, weight loss, and obvious greenish-blackish veins on the head and abdomen that only a traditional healer or spiritualist can heal [25]. *Asram* has been well reported in various studies across Ghana [18, 26–29].

This critical analysis provides an understanding of the indigenous beliefs and practices surrounding newborn fontanelles. According to these beliefs, failure of the fontanelle to close properly may allow air to enter the brain, resulting in persistent headaches, runny nose, nasal congestion, and other health issues that can persist into adulthood. As a result, traditional practices such as hot fomentation and scalp molding are used for up to two to three months after the child's birth to promote healing and closure of the fontanelles.

### Description of hot fermentation practice

A combination of five traditional practices, including hot fomentation of fontanelles, abdominal warm compress, body massage, anogenital dripping with warm water, and bathing, is performed twice daily for newborns in Ghana. This 5-in-1 procedure is typically done in the early morning and evening around 6:30 am and 4:30 pm, respectively, although the timing may vary depending on the climate. In hot weather, the newborn may be washed with non-boiled water in the afternoon for comfort [30]. Bathing is usually done as the last procedure of the five which is carried out by a female family elder who is either the grandmother, mother–in–law, sister-in-law, auntie, or trusted volunteer neighbour [31]. This sequential procedure continues until about the end of the second or third-month post-delivery when the mother is able to do it unaided herself. The person performing the procedure wraps a cloth around her waist, and often sits on a low stool, extends the legs over a rubber or metallic bath bowl or basin, and places the baby on her thighs after rolling her waist cloth backward.

**Fig. 2:**
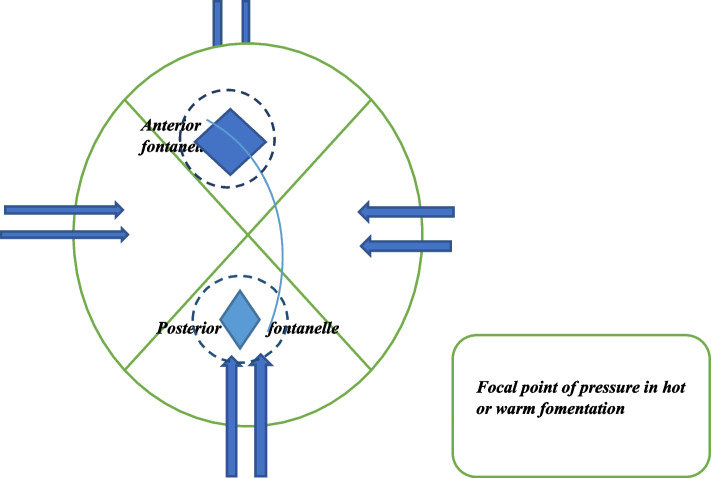
Infant Head Molding – Direction of Pressure

To perform the fontanelle fomentation and associated procedures, several items are required, including two buckets for hot and cold water, an extra bucket for mixing the water, two small towels, soap, sponge, and a moisturizing cream such as shea butter, Vaseline, baby cream, or baby oil. The fontanelle fomentation is typically the first procedure performed and lasts approximately 25 to 30 min, with the anterior fontanelle receiving about 15 min of attention due to its larger size. During the fomentation, the newborn is dressed and wrapped in a cloth, positioned face to face with the caregiver who places the baby on their closed thighs. The water temperature for fomentation is typically 70–85 °C, with visible steam. The caregiver drops the small towel into the hot water and quickly picks it up, allowing some heat to escape for about 30 s before squeezing out the excess water. The towel is then placed on the anterior fontanelle first and then rotated to the other fontanelles and along the suture lines to mold the scalp in a roundish motion. (See Fig. 2).

The fontanelle fomentation procedure typically elicits a strong reaction from newborns, who cry loudly and continuously. Once this procedure is complete, the abdominal warm compress, body massage with flexion and extension of the limbs, and ano-genital dripping with warm water are performed. Bathing from head to toe is the final step and takes approximately 15 min, after which the baby is dried and dressed. The entire 5-in-1 procedure can take up to one hour on average. If the umbilical cord has dropped, irrigation of the cord base is performed with warm water. However, if the cord has not dropped, it is kept dry and intact during the bath. This entire process is typically carried out twice a day in the bedroom or living room. During the wet or rainy season when the environmental temperature is lower than the average temperature of 23—28 °C, the neonate may be wiped clean in the evenings but will still undergo fontanelle fomentation.

### Leininger’s theoretical framework

The chosen theory for this paper is Madeleine Leininger’s Culture Care Diversity and Universality care theory [32]. This theory emphasized consideration of the patient's cultural background in order to provide the patient with culturally sensitive care. Knowing the patient's values, beliefs, and cultural practices can help provide universal care to promote patient health and well-being. This theory, therefore, emphasizes the need for healthcare providers to have a deep understanding of the patient's culture in order to provide compassionate care [33]. This theory was applicable because it ensures that nurses avoid judging or angering mothers because of this inherent practice. This suggests that we need to find innovative ways to educate mothers about the dangers involved in this practice. It, therefore, takes patience, understanding, and diligence to help mothers undo this practice.

### Dangers of newborn fontanelle fomentation practice

Fontanelle fomentation, while a commonly practiced tradition, poses potential dangers to newborns. One major concern is the risk of scalding from hot water, which can result in burns to the fontanelle. Additionally, parents applying too much pressure to the fontanelle during fomentation could lead to inflammation, internal brain tissue trauma, infection, premature fontanelle closure, and excessive molding. These risks are particularly concerning as newborn mortality trends indicate that infections are a major cause of death. Therefore, caution should be exercised when performing fontanelle fomentation to avoid potential harm to the newborn [34]. Traditionally, mothers have very little power to intervene during the fontanelle fomentation procedure of their newborns due to inherent patrilineal hegemony within the Ghanaian culture. Maternal emotional trauma is linked to the persistent cry of the newborn during the procedure which raises ethical concerns about newborn health and safety and maternal mental health over the span of 2 – 3 months, and beyond. These practices reveal the complexity of predominant culture, the lived reality of newborns, and the structural drivers of subtle but obvious health risks that impact newborns in African communities [17, 18, 20, 27, 28].

## Discussions

Indigenous practices in Ghana and Africa are predominantly passed down through oral tradition, resulting in limited information available in ethnic and professional literature. These practices are often considered cultural norms and have been in practice for many years. To reverse such behavior, it is recommended that healthcare providers exhibit care and understanding, as suggested by Madeleine Leininger. The repeated exposure of newborns to harmful indigenous practices calls for integrated healthcare and community-based approaches at all levels of newborn healthcare. Key stakeholders in newborn health, including mothers, fathers, grandmothers, mothers-in-law, sisters-in-law, grandparents, traditional birth attendants, women's groups, religious authorities, and legislative institutions, need to be engaged during the antenatal period, immediate postnatal period, and across the continuum to co-lead campaigns against harmful indigenous newborn practices [35]. These educative health campaigns can be strengthened prenatally and at birth with a focus on the family and other home care providers in newborn discharge planning [36–38]. Collaborative research related to short and long-term outcomes of NFF and other harmful indigenous newborn care practices is needed in Ghana and among African populations [39]. Newborn frontline health care providers need to emphasize the normality of the anterior fontanelle in prenatal, discharge and post-natal education sessions and examine fontanelle fomentation practices on preterm and small for age, and sick babies in home-based care.

In the current Covid-19 pandemic, healthcare access to newborns in Ghana is limited due to physical distancing as part of mobility restrictions across urban and rural communities [4]. The marginal shift from routine facility-based health care associated with Covid-19 related restrictions have heightened sub-optimal healthcare-seeking behaviours of families of newborns. This calls for feasible, sustainable and cost-effective follow up systems in newborn home-based care [40].

With considerations of the contextual limitations, indigenous practices on newborns in Ghana and other African communities are anticipated to rise during the current pandemic. Therefore, optimising the delivery of skilled home-based care on a continuum and strengthening the capacity of families in safe newborn care is needed as a matter of urgency in the current Covid-19 pandemic [4]. Health research is needed to investigate the flourishing of indigenous newborn practices during this Covid-19 pandemic. It is equally important to focus on individual behavioural changes and the impact of community beliefs on behavioural change or modifications [19].

Implications for Policy**:** Policymakers need to understand that knowing the deep-rooted traditional norms and their importance to the people is critical for educating and modifying or stopping harmful traditional practices that are rooted in predominant cultural beliefs [18, 41]. Making available educational modules in locally-friendly languages and images to families and health providers, and policies that support home care follow-ups on newborn care is important. However, stringent health policies need to be relaxed in order to bridge the gap between newborn traditional care providers and facility care providers [42]. While it is important to reduce the risks associated with harmful indigenous practices, there is a need to increase newborn advocacy and protection for newborns in culturally-dominated communities [27, 37, 41]. Whilst enlightenment campaigns and supportive child health policies are critical**,** legislation to criminalize harmful indigenous practices needs to be established and prioritized on national educational and legal agendas [20, 41, 43].

## Conclusion

The present study has explored the practice of hot fomentation, highlighted its associated risks, and proposed interventions to help mothers discontinue this practice. To effectively promote newborn care in the context of the ongoing Covid-19 pandemic, a collaborative and community-based healthcare approach that integrates family and community values, cultural beliefs, and newborn care practices is essential. This approach should be supported by evidence-based research on fontanelle fomentation, policy guidance, and the contextualization of behavioral change strategies that align with local cultural norms and national health goals. By adopting this approach, the United Nations General Assembly's Sustainable Development Goals (SDGs) to reduce the under-five mortality rate (U5MR) to at least 25 deaths per 1,000 live births can be achieved.

## Data Availability

All datasets generated during the current study are included in the published article and its supplementary files.
